# RT-CBAM: Refined Transformer Combined with Convolutional Block Attention Module for Underwater Image Restoration

**DOI:** 10.3390/s24185893

**Published:** 2024-09-11

**Authors:** Renchuan Ye, Yuqiang Qian, Xinming Huang

**Affiliations:** Department of Electronic Information Engineering, School of Ocean Engineering, Jiangsu University of Science and Technology, Zhenjiang 212003, China; gongdengdu11@163.com (Y.Q.); 19106856302@163.com (X.H.)

**Keywords:** deep learning, transformer, image restoration

## Abstract

Recently, transformers have demonstrated notable improvements in natural advanced visual tasks. In the field of computer vision, transformer networks are beginning to supplant conventional convolutional neural networks (CNNs) due to their global receptive field and adaptability. Although transformers excel in capturing global features, they lag behind CNNs in handling fine local features, especially when dealing with underwater images containing complex and delicate structures. In order to tackle this challenge, we propose a refined transformer model by improving the feature blocks (dilated transformer block) to more accurately compute attention weights, enhancing the capture of both local and global features. Subsequently, a self-supervised method (a local and global blind-patch network) is embedded in the bottleneck layer, which can aggregate local and global information to enhance detail recovery and improve texture restoration quality. Additionally, we introduce a multi-scale convolutional block attention module (MSCBAM) to connect encoder and decoder features; this module enhances the feature representation of color channels, aiding in the restoration of color information in images. We plan to deploy this deep learning model onto the sensors of underwater robots for real-world underwater image-processing and ocean exploration tasks. Our model is named the refined transformer combined with convolutional block attention module (RT-CBAM). This study compares two traditional methods and six deep learning methods, and our approach achieved the best results in terms of detail processing and color restoration.

## 1. Introduction

Underwater image restoration is a task that aims to restore high-quality images through removing degradations such as noise, blur, and chromatic aberration. In the early stages, due to limitations in computing power and image-sensing technology, underwater image processing mainly relied on simple image enhancement techniques and basic image analysis methods. Most of the work focused on using optical filters [1] and manual methods to improve the visual quality of images.

With the further improvement of computational processing power and the development of new algorithms, physical models [2,3] began to be used to more accurately restore the true colors and details of images. These models restore images through simulating the attenuation and scattering of light in water. After achieving notable progress with physical models, researchers started exploring the application of statistical and machine learning methods in underwater image processing. Using image statistical features for dehazing and enhancement became the main focus during this stage. Underwater image restoration technology gradually shifted from prior-based strategies to deep learning-based methods [4,5,6,7,8,9,10,11,12].

In recent years, the rapid development of deep learning has brought new opportunities for underwater image processing. Deep learning architectures have been extensively employed for tasks such as the dehazing, enhancement, and restoration of underwater images. Through training on large-scale data, deep learning methods can automatically extract image features, significantly improving processing effectiveness. However, CNNs have limitations in establishing global dependencies and multi-scale information, while GANs face challenges in integrating global information. Therefore, more advanced technologies have been introduced, such as transformers [12,13], making significant contributions to high-level vision tasks. Nonetheless, transformer technology still faces the following challenges:

1. Insufficient ability to handle fine local details, which is crucial for underwater image restoration and enhancement;
2. Poor independence of color channels, limiting the ability to perceive color deviations.

In this study, we designed a transformer-based encoder–decoder backbone network for underwater image restoration. In order to address the first issue mentioned above, we proposed a transformer-based model by introducing and improving the dilated transformer block (DTB) [14]. In the self-attention mechanism, through concatenating Q and K in the channel dimension and performing convolution operations, more contextual information and feature interactions can be captured, enhancing feature representation capabilities. Next, we embedded the local and global blind-patch network (LG-BPN) [14] in the bottleneck layer. LG-BPN simultaneously handles local and global features, capturing local details and global dependencies, allowing the network to better preserve details and textures. Specifically, in local feature information processing, we first use a 9 × 9 dense sampling patch mask convolution (DSPMC) to capture local detail information through the dense sampling of neighboring pixels, followed by dilated convolution to expand the receptive field. In global feature information processing, we first use a 21 × 21 DSPMC module to capture global information, followed by the dilated transformer block (DTB) to model global dependencies. Finally, local and global image features are fused and processed through convolution to generate the image. This approach offers the following three advantages:

1. It preserves the model’s capability to capture long-range dependencies;
2. Combining local details and global dependencies avoids detail loss and texture destruction;
3. It utilizes more neighboring pixels when processing images, achieving finer handling of small, local details and improving the accuracy of detail restoration.

In order to address the color issues in underwater image restoration, we utilized the multi-scale convolutional block attention module (MSCBAM) to connect symmetric encoder and decoder feature blocks. CBAM is a widely used spatial channel attention mechanism that enhances the model’s ability to independently perceive different color channels by applying attention mechanisms to both spatial and channel dimensions. Specifically, CBAM first extracts features for the channel dimension through global average pooling and global max pooling and then generates the channel attention map using a shared multilayer perceptron (MLP). Channel attention can identify the channels that need to be emphasized during color restoration, effectively improving color handling by emphasizing important color features and suppressing less important ones. This enables CBAM to address color processing issues, making the colors of the restored images more natural and consistent. The primary contributions of this study are outlined as follows:

- We designed a multi-scale refined dilated transformer block model which applies RDTB at different scales. This enables the network to capture detailed information over a larger range, enhancing detail recovery capability and enhancing image visual quality. The multi-scale application allows the network to capture richer contextual information, thereby better modeling the global dependencies and local structures in the images.
- In the bottleneck layer, we embedded a self-supervised method (local and global blind-patch network) that combines the advantages of RDTB in handling underwater image details. This allows the model to better understand global contextual information while utilizing local details and global interactions to restore fine structures in the images.
- We used the multi-scale convolutional block attention module (MSCBAM) to connect symmetric features, capturing color feature information at different scales. This effectively removes issues related to color processing, such as chromatic aberration and color bias.

## 2. Related Works

Underwater image restoration is extremely important for the development of ocean engineering [15] and underwater robotics [16]. Underwater image-restoration technology has gradually matured and, at present, can be broadly categorized into two types: traditional methods [2,3,17,18,19] and deep learning-based methods [4,5,6,7,8,9,10,11].

### 2.1. Underwater Image Restoration Based on Traditional Methods

Traditional underwater image restoration methods can be mainly divided into physics-based models [2,3] and non-physics-based models [17,18,19].

Physics-based models are a common approach for processing underwater images. The basic idea is to model the light propagation and imaging process in detail, taking into account physical phenomena such as absorption and the scattering of light in water, thereby improving the clarity and quality of underwater images. Akkaynak et al. [2] proposed a revised underwater image formation model, which considers the characteristics of light absorption and scattering and introduces a new correction method to improve the quality of underwater images. Chiang et al. [3] proposed an underwater image-enhancement method based on wavelength compensation and dehazing, which corrects color distortion and haze effects in images through a physical model. Enhancement methods based on physical models rely on the prior knowledge of the model. However, prior knowledge can often lead to significant estimation bias in different underwater scenarios. Moreover, fundamental parameters such as underwater depth and light propagation coefficients are difficult to obtain.

Non-physics-based models for processing underwater images rely more on statistical characteristics, prior knowledge, and data-driven approaches. These methods process and enhance images by observing and learning the statistical properties within the images. Galdran et al. [17] proposed an automatic underwater image-restoration method based on the red channel by using statistical prior information to improve the color and clarity of images. Fu et al. [18] introduced a single underwater image-enhancement method based on Retinex theory, enhancing the visual effects of images through histogram equalization. He et al. [19] proposed a “dark channel prior” for removing haze from images, demonstrating its potential in underwater image enhancement. However, non-physics-based models do not consider the physical degradation mechanisms of underwater images.

### 2.2. Underwater Image Restoration Based on CNNs

CNNs have attained considerable success in processing underwater images. CNN models can automatically learn and capture important feature information from the input underwater images, improving image quality. Various CNN-based underwater image-processing networks have been proposed. Li et al. [4] introduced UWCNN, a convolutional neural network specifically designed for underwater image restoration, which repairs images by learning the end-to-end mapping of degraded images to clear images. Tao et al. [5] proposed a method that combines CNNs and bright channel prior techniques to enhance low-light images, effectively improving image visibility while preserving color details. Wu et al. [6] proposed a new, two-stage underwater image convolutional neural network (UWCNN-SD) based on structure decomposition for underwater image enhancement. However, CNNs primarily rely on local receptive fields to extract image features, which limits their ability to capture long-range dependencies and global features. They perform poorly when handling tasks that require global context. The feature-extraction method of CNNs is relatively fixed, making it difficult to adapt to the diverse needs of different tasks.

### 2.3. Underwater Image Restoration Based on Generative Adversarial Networks

In a GAN, the goal of the generator is to create images that are as realistic as possible, whereas the discriminator’s role is to tell real images apart from generated ones. Underwater image restoration methods based on GANs also have very broad applications. Li et al. [7] proposed an unsupervised GAN called WaterGAN, which uses images from the air and the corresponding generated images that resemble real underwater images as the training dataset. Through end-to-end network training, this method corrects the colors of underwater images and can achieve the real-time color correction of single underwater images. Islam et al. [8] proposed an underwater image-enhancement method based on CycleGAN, which learns a transformation model from unpaired image datasets through unsupervised learning, improving image quality and visual perception. Fabbri et al. [9] introduced an underwater image-enhancement method based on GANs, which generates more realistic images through adversarial training, significantly enhancing the clarity and color restoration of underwater images.

### 2.4. Underwater Image Restoration Based on Transformer

Transformers were initially used for natural language processing (NLP); however, due to their powerful self-attention mechanisms and parallel processing capabilities, they have slowly made their way into the field of computer vision in recent years. Transformer models can capture long-range dependencies and global features when handling image tasks, which is particularly important in underwater image processing, where complex scenes and multiple degradation factors coexist. At present, more and more researchers are incorporating transformers into underwater image restoration models. Zamir et al. [9] significantly improved the performance and efficiency of image restoration by enhancing the attention mechanisms in transformers. Wang et al. [11] proposed a general U-shaped transformer model that significantly improved image-restoration effects through introducing multi-scale feature-extraction and fusion mechanisms. The self-attention mechanism in transformers is highly effective at identifying long-range dependencies and global features, effectively modeling these complex relationships.

## 3. Materials and Methods

### 3.1. Network Architecture

**Figure 1:**
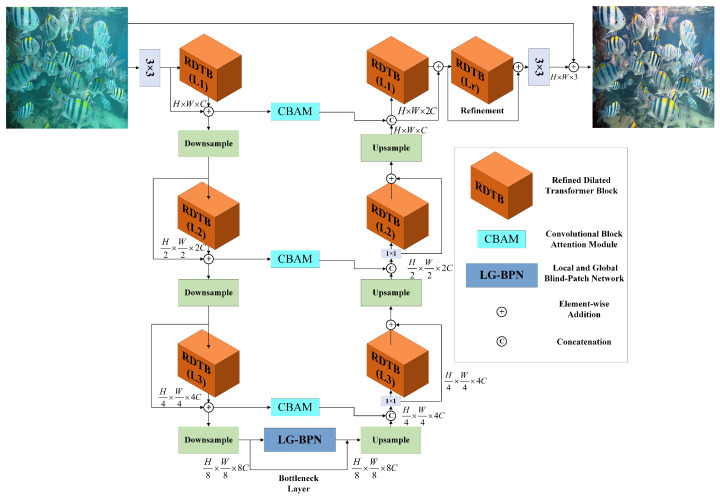
The diagram illustrates the complete architecture of the RT-CBAM model. This model consists of a multi-scale hierarchical design of refined dilated transformer blocks. It also includes convolutional block attention module to enhance feature representation capabilities and a local and global blind-patch network for efficient feature extraction and fusion.

Figure 1 presents the overall structure of the suggested RT-CBAM model. It includes a U-net-structured backbone network, the channel attention mechanism CBAM, and the local and global blind-patch network (LG-BPN) for efficient feature extraction and fusion. The final output image is obtained through this model.

Overall Pipeline. Given an initial image, $X_{i}\in R^{H\times W\times 3}$, the RT-CBAM model first acquires the shallow features of the input image through a 3 × 3 convolution. The shallow feature mapping is denoted as $F_{S}\in R^{H\times W\times C}$, where H×W represents the spatial dimensions, and *C* represents the number of channels. Next, a three-level symmetrical encoder–decoder progressively converts the shallow features, $F_{S}$, into deep features, $F_{d}\in R^{H\times W\times 2C}$. Each scale of the encoder–decoder is composed of multiple refined dilated transformer blocks, with the number of blocks increasing progressively to refine feature maps while maintaining model efficiency. The shallow features $F_{S}$ are initially processed by the encoder part, where after three scales of the encoder, downsampling is performed at each scale, reducing the spatial size while increasing the channel capacity, with feature mappings denoted as $F_{E-i}\in R^{\frac{H}{2^{i}}\times \frac{W}{2^{i}}\times 2^{i}C}\left(i=1,2,3\right)$. Correspondingly, the decoder part performs upsampling, progressively restoring the spatial size while reducing the channel capacity, with feature mappings denoted as $F_{D-i}\in R^{\frac{H}{2^{i}}\times \frac{W}{2^{i}}\times 2^{i}C}\left(i=2,1,0\right)$. In order to aid in the restoration of color information, the encoder features are concatenated with the decoder features in the channel dimension using the multi-scale convolutional block attention module (MSCBAM), followed by a 1 × 1 convolution to halve the number of channels. In order to better integrate the low-level features from the encoder with high-level features and maintain the texture and structural details of the image, the topmost layer of the decoder connects to the encoder features without convolution, thus preserving the integrity of detail information. The bottleneck layer that connects the encoder and decoder uses the LG-BPN to capture both global and local information, further refining feature maps and enhancing the model’s expressive capabilities. The deep features $F_{d}$ obtained from the decoder at high spatial dimensions are further enriched during the refinement stage. At each scale, we add residual connections, allowing the model to effectively utilize information across different scales. Finally, a 3 × 3 convolution is used to generate the residual image, $R\in R^{H\times W\times 3}$, which is added to the initial image to obtain the restored image $X_{o}=X_{i}+R$.

### 3.2. Refined Dilated Transformer Block

In the traditional transformer, the self-attention mechanism performs well in capturing long-range dependencies but needs improvement in handling local details and high-frequency features. In order to process underwater images containing complex and fine structures, we made some modifications to the attention mechanism in the transformer block. Our proposed refined dilated transformer block consists of two core components: the improved multi-head self-attention mechanism layer and the feed-forward network layer. Through concatenating the query (Q) and key (K) of each head in the multi-head attention mechanism along the channel dimension, we capture more contextual information. Subsequently, a 1 × 1 convolution is used to facilitate information interaction between Q and K. Then, the resulting Q–K interaction information is element-wise multiplied by the output of the self-attention mechanism, giving each head a corresponding scaling factor to correct the attention, thereby enhancing the accuracy of the attention mechanism. This improvement not only captures more contextual information but also significantly enhances the expressive capability of the attention mechanism and the overall performance of the model by promoting information interaction and accurately correcting attention. The proposed attention mechanism not only introduces dilated convolutions to enhance local spatial features within each channel of the image but also maintains linear computational complexity. This makes it equally suitable for processing high-resolution images, ensuring the model’s efficiency and generalization capability.

**Figure 2:**
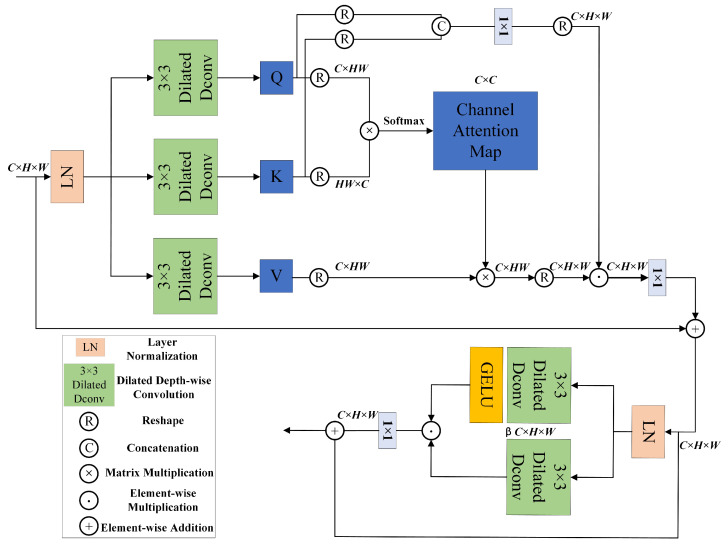
The overall structure of the enhanced transformer module comprises two components: the self-attention mechanism and the feed-forward network. Enhancing the self-attention mechanism notably boosts the feature representation capability of this module.

Figure 2 is divided into two layers, the self-attention layer and the feed-forward network layer.

First, for the self-attention layer, given an input tensor $X\in R^{H\times W\times C}$, and after layer normalization, this layer is introduced into a dilated 3 × 3 depth-wise convolution to encode the channel-wise spatial context and generate query (Q), key (K), and value (V) projections. Their matrices can be represented as $Q=C_{d}^{Q}\left[LN\left(X\right)\right]$, $K=C_{d}^{K}\left[LN\left(X\right)\right]$, and $V=C_{d}^{V}\left[LN\left(X\right)\right]$, where $C_{d}^{Q}\left(\cdot \right)$, $C_{d}^{K}\left(\cdot \right)$, $C_{d}^{V}\left(\cdot \right)$ represents dilated 3 × 3 depth-wise convolution and represents layer normalization. Next, we reshape Q and K, use their dot product to obtain channel interactions, and generate the attention map $X_{A}\in R^{C\times C}$, where *C* represents the number of channels. Meanwhile, within the channel dimension, we concatenate the reshaped Q and K in each head to capture richer contextual information, thereby helping the model better understand the overall structure and relationships of the input sequence. Subsequently, a 1 × 1 convolution is used to perform a linear transformation between the channels of Q and K, enabling a higher-dimensional fusion and enhancing the information interaction between them. Finally, the Q–K interaction information is element-wise multiplied by the output of the attention layer to achieve attention correction. The self-attention layer can be represented as

(1) Attention(Q,K,V)=V×Softmax(QK)

(2) $$X^{\prime }=C_{p}\left[C_{p}^{QK}⊙\mathrm{Attention}\left(Q,K,V\right)\right]+X$$

where *X* and $X^{\prime }$ represent the input and output feature maps, respectively; $C_{p}^{\left(\cdot \right)}$ represents 1 × 1 point-wise convolution; $Q\in R^{C\times HW}$, $K\in R^{HW\times C}$, and $V\in R^{C\times HW}$.

Second, we introduce the gated-dconv feed-forward network (GDFN) [10] to replace the traditional feed-forward (FN) network [20]. Through the gating mechanism, information flow is controlled throughout the hierarchical structure, allowing each layer to focus on details that complement other layers. In the feed-forward network layer, the output of the attention layer is first-layer normalized. Then, the normalized output is split into two parallel paths, with each path introducing a dilated 3 × 3 depth-wise convolution to encode the channel-wise spatial context, thus more efficiently extracting local image structures. The outputs of the two paths are then element-wise multiplied, with the output of one path processed through the GELU (Gaussian error linear unit) activation function, forming a gating unit. This gating unit achieves complex nonlinear transformations of the features, thereby enhancing the model’s expressive capability and performance. The channel expansion factor in the GDFN is 2.66 (β=2.66). When given an input tensor, $X^{\prime }\in R^{H\times W\times C}$, for the feed-forward network layer, its expression is as follows:

(3) $$G^{1}=C_{d}^{1}\left[LN\left(X^{\prime }\right)\right]$$

(4) $$G^{2}=C_{d}^{2}\left[LN\left(X^{\prime }\right)\right]$$

(5) $$Y=GELU\left(G^{1}\right)⊙G^{2}+X^{\prime }$$

where ⊙ represents element-wise multiplication; $C_{d}^{1}\left(\cdot \right)$ and $C_{d}^{1}\left(\cdot \right)$ represent dilated 3 × 3 depth-wise convolution. In summary, by introducing the gating mechanism, GDFN can control information flow, enabling deeper layers in the network hierarchy to concentrate on finer image details. Combined with our proposed improved self-attention mechanism, the GDFN can handle high-resolution images while maintaining linear complexity, making it suitable for large-scale image restoration tasks and providing the model with strong applicability and generalization capabilities.

### 3.3. Loss Function

The comprehensive loss function of the RT-CBAM model, $L_{\mathrm{total}}$, includes both the generator loss function, $L_{G}$, and the discriminator loss function, $L_{D}$.

The generator combines three loss functions: the pixel loss $L_{\mathrm{pixel}}$, which calculates the mean squared error at the pixel level to ensure the consistency of image details; the structural loss, $L_{\mathrm{MS}-\mathrm{SSIM}}$, which employs the multi-scale structural similarity index to assess the structural resemblance between the generated image and the actual image, and the $L_{1}$ loss function for calculating absolute discrepancy. The generator loss function is expressed as follows:

(6) $$L_{G}=\alpha \times L_{\mathrm{pixel}}+\beta \times L_{\mathrm{MS}-\mathrm{SSIM}}+\lambda \times L_{1}$$

where α, β, and λ are hyperparameters, which were set to 0.01, 100, and 10, respectively, in this experiment. For the discriminator, the adversarial loss is used, which is achieved by calculating the mean squared error (MSE) between the images generated by the generator and the real images.

(7) $$L_{D}=L_{\mathrm{GAN}}$$

Combining the pixel loss, L1 loss [12], MS-SSIM loss [21], and adversarial loss, each loss function targets different aspects of image restoration. The comprehensive use of these losses can generate high-quality images that are rich in detail and structurally consistent while ensuring the consistency and effectiveness of the training process. This combined strategy [12] enables the image-restoration model to perform better in practical applications. In conclusion, the overall loss function of the model is

(8) $$L_{\mathrm{total}}=L_{G}+L_{D}=L_{\mathrm{GAN}}+\alpha \times L_{\mathrm{pixel}}+\beta \times L_{\mathrm{MS}-\mathrm{SSIM}}+\lambda \times L_{1}$$

## 4. Experiments and Analysis

In this section, we begin by presenting the training specifics and experimental configuration of the RT-CBAM model. Subsequently, we compare our underwater image-restoration method with cutting-edge methods on existing underwater datasets. Finally, we perform a sequence of ablation experiments to validate the impact of each component of the model.

### 4.1. Details of the Experiment

We used the PyTorch 1.13.1 framework and implemented RT-CBAM model training on an NVIDIA RTX 4090. The training utilized the Adam optimizer, starting with a learning rate of $5\times 10^{-5}$. The model was trained for 300 epochs with a batch size of 2.

### 4.2. Experimental Set-Up

In this section, we summarize the experiments conducted to evaluate the RT-CBAM model’s performance in restoring underwater images. Our datasets include the LSUI Dataset [13], EVUP Dataset [22], UIEB Dataset [23], Seathru [24], and the RUIE Dataset [25], covering various underwater scenes with different water environments, brightness conditions, and target classes to promote variety. The datasets were arbitrarily segmented into two sections for training and evaluation. The training set, Train-L, comprised 3879 pairs of underwater images from LSUI and 1600 pairs from EVUP. The test set was constructed using both reference-based and non-reference-based benchmarks. The full-reference test set was divided into two groups: one group, Train-L400, randomly selected 400 pairs from the remaining LSUI data; the other group, Test-E120, comprises 80 pairs from EVUP and 40 pairs from UIEB. The non-reference test set was also divided into two groups. One group, Test-U60, consisted of images from UIEB and RUIE; the other group, Test-Seathru, was composed of images from Seathru.

In order to enrich the variety of the training dataset, we employed data augmentation techniques, such as cropping and rotation. All images were standardized to a consistent size of 256 × 256 pixels as inputs to the network, with pixel values normalized to the range [0, 1]. This preprocessing method ensured that the model could better adapt to different image features, improving the performance and robustness of underwater image restoration.

We benchmarked the proposed model against several cutting-edge methods, including deep learning methods (Waternet [22], U-Trans [13], FUnIE-Gan [23], UGAN [9], STSC [26], and RAUNE-Net [27]), physics-based methods (UDCP [19]), and visually driven prior methods (Retinex-based [18]).

In this study’s experiments, we used various evaluation metrics to comprehensively assess the model’s performance. For the reference-based test dataset, we employed four different evaluation metrics: PSNR [28], SSIM [29], MAE [30], and LPIPS [31]. PSNR measures the quality of image reconstruction, with higher PSNR values indicating that the restored image is closer to the reference image. SSIM evaluates the similarity in brightness, contrast, and structure, closely approximating human visual perception. MAE measures the mean absolute error at the pixel level, with smaller values indicating less error. LPIPS calculates the perceptual differences between images using features extracted by deep neural networks, with lower LPIPS values indicating higher perceptual quality. For the non-reference-based test dataset, we used three evaluation metrics: UIQM [32], UCIQE [33], and NIQE [34], which collectively consider the contrast, color, and clarity of underwater images.

### 4.3. Network Architecture Assessment

**Figure 3:**
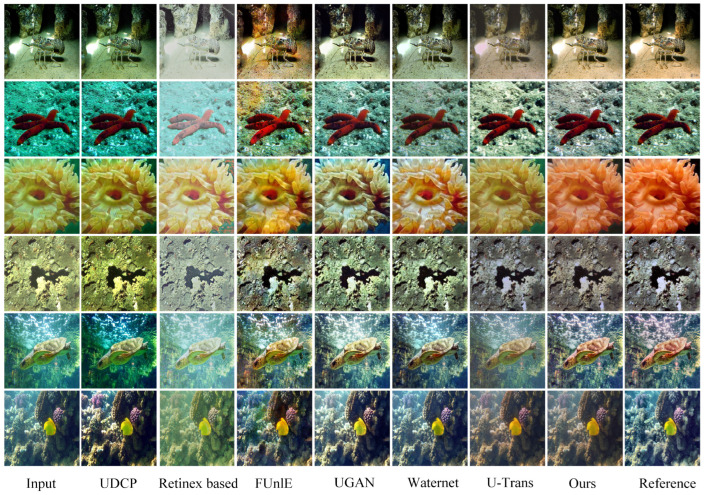
Visual comparisons of restoration results sampled from the Test-L400 and Test-E120 datasets are shown from left to right: original underwater image, UDCP [19], Retinex-based [18], FUnIE-GAN [23], UGAN [9], Waternet [22], U-Trans [13], our proposed RT-CBAM, and the reference image.

Full-reference evaluation: The evaluation was conducted using the Test-L400 and Test-E120 datasets. The numerical results and visual assessments are consolidated in Table 1 and Figure 3. Compared with two traditional methods and six deep learning-based methods, the proposed RT-CBAM model attained the highest performance in PSNR and SSIM metrics alike. Our model’s restoration results are most similar to the reference images, with reduced color artifacts and clearer image textures. The potential limitations of the six deep learning-based methods are analyzed below. The UDCP and Retinex-based models exhibit significant issues with color and lighting processing, showing severe color biases with noticeable blue or green tints, making objects appear unnatural. UGAN and FUnIE-GAN retain blue or green artifacts around object edges and exhibit unnatural colors, lacking detail-handling capabilities. Waternet’s detail recovery is incomplete, showing a green color bias. U-Trans, due to its complex global feature extraction method, can cause local detail distortion, especially when processing high-resolution images, making local textures appear unnatural or distorted. Furthermore, we used MAE and LPIPS for full-reference evaluation. As shown in Table 1, our proposed method achieved the highest scores, indicating that the restored images are visually closer to the reference images in terms of perceptual quality.

**Figure 4:**
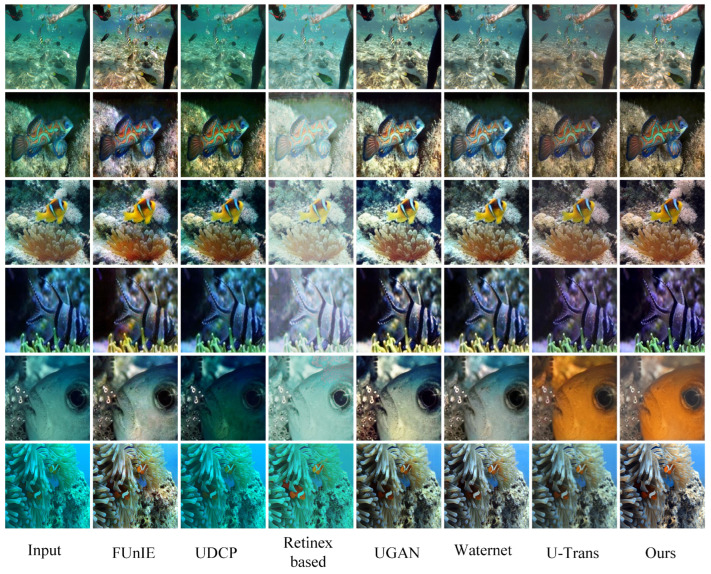
The restoration results sampled from Test-U60 are visually compared and displayed, with images presented from left to right as follows: original underwater image, FUnIE [23], UDCP [19], Retinex-based [18], UGan [9], WaterNet [22], U-Trans [13], and the proposed RT-CBAM.

**Figure 5:**
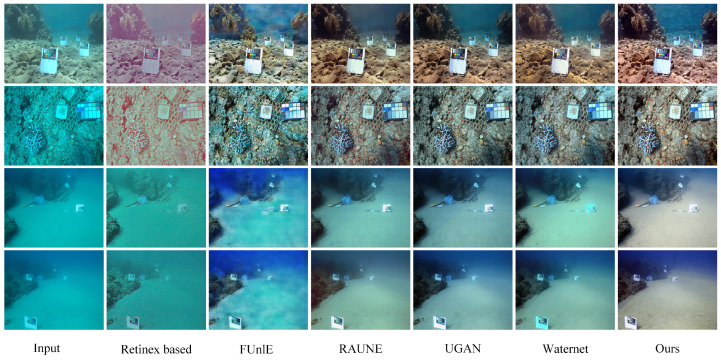
A visual comparison of the restoration results sampled from Test-Seathru is shown, with selected images being high-resolution (1280 × 1280 pixels). The images, from left to right, are the original underwater image, FUnIE [23], UDCP [19], Retinex-based [18], UGan [9], WaterNet [22], U-Trans [13], and the proposed RT-CBAM.

Non-reference evaluation: The evaluation was conducted using the Test-U60 and Test-Seathru datasets. The statistical findings and visual comparisons are outlined in Table 2 and Figure 4 and Figure 5. According to the experimental results, we found that the UDCP model significantly improved contrast, but the color bias towards blue caused the images to have an unnatural cold tone. Detail recovery was also less than ideal, with many details submerged in the high contrast. The Retinex-based model, while improving brightness to some extent, exhibited severe color bias issues, with images presenting an unnatural blue-green tint and blurred details. FUnIE-GAN still had color deviations throughout the images, particularly in vibrant areas. The UGAN model improved color processing but retained some blue or green artifacts, especially in detail-rich regions, with overall deficiencies in color and detail handling. Waternet improved image clarity to some extent but failed to restore colors accurately, resulting in noticeable color bias. The U-Trans model exhibited local detail distortion and overall darkness. In our proposed method, we improved the self-attention module of the transformer to more accurately extract image details and textures. We also introduced multi-scale CBAM into the model to enhance attention to color channels and detail textures, thereby optimizing the image’s texture and color.

### 4.4. Comparative Assessment of Detailed Restoration

**Figure 6:**
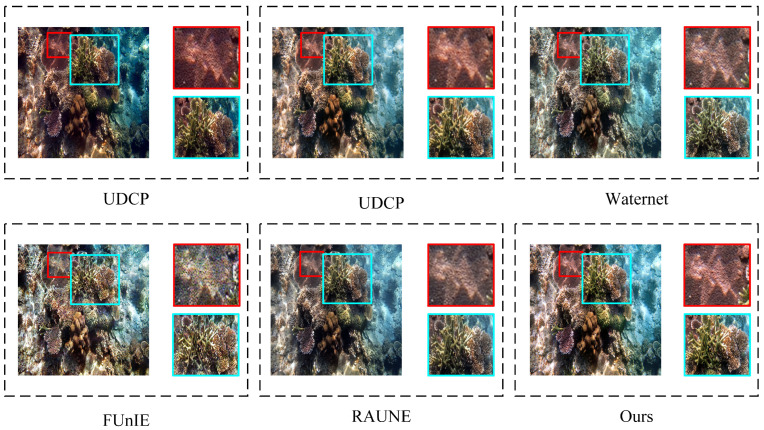
Qualitative comparison on the UIEB dataset. The restoration results obtained by our algorithm exhibit more pleasing contrast and more precise textures.

**Figure 7:**
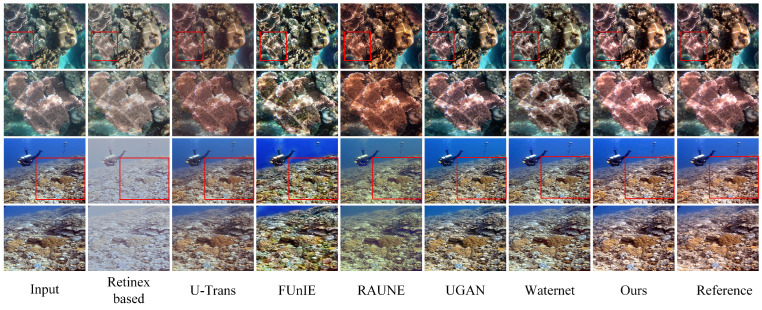
Evaluation of detail restoration in high-resolution images. From left to right, the images are the original underwater image, Retinex-based [18], U-Trans [13], FUnIE-Gan [23], RAUNE-Net [27], UGan [26], Waternet [22], and our proposed RT-CBAM.

By evaluating the effectiveness of eight distinct underwater image-restoration methods on the Test-E120 dataset, the effectiveness of our method in detail restoration is demonstrated. The experimental results are shown in Figure 6 and Figure 7. UDCP achieves better visual effects compared to some deep learning methods, but its overall darkness affects the restoration of detailed textures. The Retinex-based model, although enhancing local contrast in certain areas, results in generally darker images with unnatural color restoration, making the images look dull and less vivid. The U-Trans model tends to give images an unnatural reddish hue, which affects the realism of the images. The FUnIE model imparts a yellowish tint to the images, and while the brightness is improved, the color deviation affects the visual effect. The RAUNE model, in its attempt to correct colors and add details, often introduces an excessive amount of blue, leading to color imbalance. The UGAN model’s overly saturated colors cause detail distortion. While the WaterNet model improves brightness and contrast, it still fails to adequately restore details clearly, resulting in a slight lack of image clarity.

As shown in Figure 6 and Figure 7, we compared the performance of Retinex-based, U-Trans, FUnIE, RAUNE, UGAN, Waternet, and our proposed model in terms of detail restoration. From the zoomed-in regions, it can be observed that UDCP exhibits relatively coarse texture details, and RAUNE shows noticeable noise in the details. Retinex-based and U-Trans models have significant blurring issues in texture processing, and UGAN and Waternet display unnatural color blotches or distortions in some texture details. In contrast, our model demonstrates clearer performance in local details, accurately restoring complex texture structures and significantly reducing noise, resulting in more natural and refined overall textures.

### 4.5. Color Reproduction Assessment

**Figure 8:**
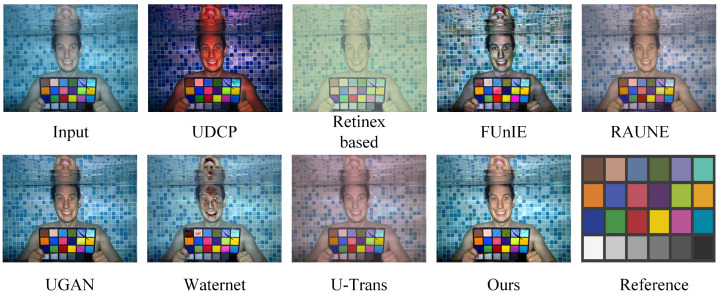
Visual comparison and evaluation of color restoration performance selected from the Color-Checker7 dataset.

Through evaluating the effectiveness of eight distinct underwater image restoration methods on the Color-Checker7 dataset, we demonstrate the precision of our approach in color correction. The Color-Checker7 dataset includes seven underwater images taken by different cameras, each containing a standard color checker with 24 different color patches. These patches have known reference values under various lighting conditions. We used CIEDE2000 [35] to compare the differences between the known reference values and the restoration results. The experimental outcomes are shown in Table 3 and Figure 8.

According to the data in Table 3, our method shows the best performance in color difference for cameras such as Pentax W60, Pentax W80, Fuji Z33, Panasonic TS1, and Olympus T6000, achieving the lowest color difference values. Additionally, our method ranks among the top in average scores. These results confirm the superior performance of our method in underwater color correction.

As shown in Figure 8, our proposed method exhibits the best visual effect among all methods, indicating its superior color correction capability. In contrast, UDCP, FUnIE, RAUNE, and U-Trans exhibit varying degrees of color cast, making them appear unnatural. FUnIE and Waternet suffer from severe color distortion, while UGAN is overall too dark, affecting the visual experience.

### 4.6. Ablation Experiments

For the ablation study, we used the Test-L400, Test-E120, and Test-U60 datasets. We considered three factors: the improved transformer, CBAM, and LG-BPN. The model in this experiment was trained using the Train-L dataset, and the baseline model was the Restormer model proposed by Syed Waqas Zamir et al. [10]. As shown in Table 4, our complete model achieved the best quantitative performance across all three test datasets, obtaining the highest scores in PSNR, SSIM, UIQM, and UCIQE metrics. This indicates the superiority of our proposed method.

**Figure 9:**
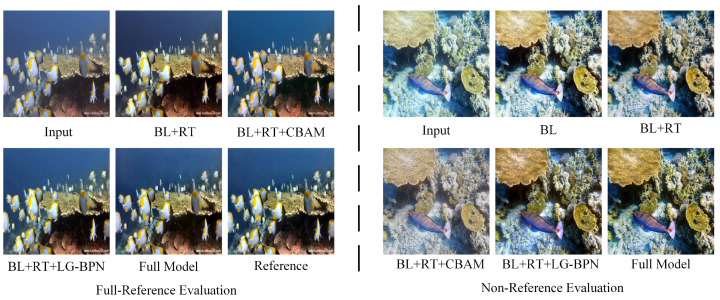
Visual comparison of the ablation study sampled from Test-E120 and Test-U60. The left side represents full-reference evaluation, and the right side represents no-reference evaluation.

As shown in Figure 9, the results of the complete enhanced model exhibit the highest PSNR and the best visual effects. Compared to the baseline model (BL), the BL + RT model shows improvements in local texture details and reduces some color casts. The addition of the multi-scale channel attention mechanism further enhances color processing, making the colors in the BL + RT + CBAM model more vivid. Given the complex texture details in underwater images, LG-BPN was introduced to address this issue. The experimental results show that BL + RT + LG-BPN achieves even better detail handling. These ablation experiments demonstrate that each of the three modules studied plays a specific role in the enhancement process, with each fulfilling its function. The overall combination improves the network’s performance and adapts to different underwater image scenarios.

## 5. Discussion

In this study, we proposed an efficient underwater image-restoration model based on a transformer architecture, referred to as RT-CBAM. Our approach introduced several key enhancements over existing state-of-the-art models. First, we incorporated innovative designs into the self-attention mechanism to enhance the extraction of detailed features, addressing the limitations of conventional transformers in handling fine local details. This improvement allowed our model to more effectively recover intricate patterns and textures, which are often lost in underwater imagery due to the scattering of light underwater.

Second, we utilized a multi-scale convolutional block attention module (CBAM) to link the encoder and decoder. This design enabled the network to better perceive and correct color distortions, a common issue in underwater imagery caused by the absorption and scattering of different wavelengths of light. By addressing these challenges, RT-CBAM achieved superior color restoration, significantly improving the quality of the processed images compared to existing methods.

Furthermore, the integration of the local and global blind-patch network (LG-BPN) in the bottleneck layer allowed for the effective aggregation of global and local features at a highly abstracted level. This architecture enhanced the adaptability of our model to diverse underwater environments, making it robust across a wide variety of image conditions, including those with extreme visibility issues. The combination of these innovations resulted in a model that not only preserves local details but also optimizes global feature extraction, providing a comprehensive solution for underwater image restoration.

When evaluated against existing state-of-the-art models, RT-CBAM demonstrated significant improvements in both structural similarity index measure (SSIM) and peak signal-to-noise ratio (PSNR) scores. These metrics highlighted the model’s superior performance in detail recovery and color correction, establishing it as a potential new benchmark for underwater image restoration tasks.

Looking forward, several avenues for future research could be explored. First, we plan to deploy RT-CBAM onto underwater robotic systems to enable real-time image processing for ocean exploration tasks. This application would further demonstrate the model’s practical utility in real-world scenarios. To facilitate this, we intend to optimize the model’s parameters and streamline its architecture to reduce computational overhead, thereby improving both deployment efficiency and real-time processing capabilities. Reducing the model’s reliance on large-scale datasets for training while maintaining performance across a range of underwater environments is another key challenge that we aim to address.

In conclusion, while our proposed RT-CBAM model has proven to be a significant advancement in underwater image restoration, further refinements and optimizations are necessary to fully realize its potential in real-time and large-scale applications. Our work not only contributes to advancing the current technology but also provides a practical tool for the underwater robotics community, offering a solution that balances high performance with real-world applicability.

## Figures and Tables

**Table 1:** We tested different models on the Test-L400 and Test-E120 datasets for comparison. The scores for PSNR, SSIM, MAE, and LPIPS are presented, with the highest values highlighted in bold red.

Methods	Test-L400				Test-E120			
	PSNR	SSIM	MAE	LPIPS	PSNR	SSIM	MAE	LPIPS
UDCP [19]	14.754	0.687	0.077	0.275	13.821	0.62	0.092	0.353
Retinex-based [18]	15.336	0.698	0.071	0.223	14.763	0.734	0.091	0.293
FUnIE-Gan [23]	21.674	0.845	0.065	0.147	18.984	0.767	0.089	0.243
Ugan [9]	24.488	0.891	0.038	0.099	17.531	0.752	0.105	0.261
STSC [26]	22.323	0.742	0.053	0.094	18.993	0.752	0.093	0.265
Waternet [22]	24.765	0.861	0.038	0.086	19.294	0.791	0.081	0.235
RAUNE-Net [27]	25.545	0.902	0.037	0.08	25.015	0.857	0.044	0.152
U-Trans [13]	24.634	0.894	0.042	0.106	25.053	0.831	0.048	0.112
ours	**28.457**	**0.942**	**0.012**	**0.066**	**27.746**	**0.912**	**0.021**	**0.086**

**Table 2:** The models were evaluated on the Test-U60 and Test-Seathru datasets separately. The values for the top scores are highlighted in bold, with the highest scores from deep learning methods indicated in red.

Methods	Test-U60			Test-Seathru		
	UIQM	UCIQE	NIQE	UIQM	UCIQE	NIQE
input	4.722	0.514	5.463	1.810	0.481	6.970
UDCP [19]	5.962	0.613	4.809	3.848	**0.671**	6.726
Retinex-Based [18]	6.731	**0.668**	4.659	3.316	0.581	6.887
FUnIE-Gan [23]	5.338	0.590	5.021	2.898	0.540	4.511
Ugan [9]	6.358	0.578	5.952	3.193	0.551	3.904
STSC [26]	6.213	0.593	5.802	2.911	0.540	6.065
Waternet [22]	6.399	0.580	5.281	3.597	0.567	3.826
RAUNE-Net [27]	5.943	0.583	5.996	3.312	0.558	5.939
U-Trans [13]	6.309	0.557	5.211	3.158	0.543	4.836
ours	**6.822**	0.603	**4.731**	**4.691**	0.598	**3.241**

**Table 3:** Color difference comparison based on CIEDE 2000 on the Color-Checker7 dataset. The values for color difference are provided, with the best scores highlighted in red.

Methods	Pen W60	Pen W80	Can D10	Fuj Z33	Oly T8000	Oly T6000	Pan TS1	Avg
Input	12.224	18.313	14.325	17.724	16.985	13.703	14.352	15.375
UDCP [19]	12.881	15.562	12.774	21.385	18.402	14.972	18.771	16.392
Retinex based [18]	14.201	15.502	13.685	16.066	13.137	17.613	18.501	15.529
FUnIE-Gan [23]	11.351	14.283	11.774	16.322	16.061	12.275	17.484	14.221
Ugan [9]	10.115	10.977	10.803	15.521	11.807	11.524	16.151	12.414
STSC [26]	11.078	10.664	9.021	15.473	12. 871	10.801	16.174	12.297
Waternet [22]	9.592	11.082	10.134	14.044	11.154	10.403	12.084	11.213
RAUNE-Net [27]	9.675	12.156	9.993	11.586	12.386	10.017	10.235	10.864
U-Trans [13]	9.781	9.899	9.237	13.208	13.786	9.903	9.922	10.819
ours	7.231	9.513	9.501	11.074	11.019	9.622	7.473	9.374

**Table 4:** Scores from the ablation study on the Test-L400, Test-E120, and Test-U60 datasets, with the highest scores highlighted in bold.

Models	Test-L400		Test-E120		Test-U60	
	PSNR	SSIM	PSNR	SSIM	UIQM	UCIQE
BL	21.166	0.814	20.632	0.804	6.143	0.547
BL + RT	23.587	0.848	22.323	0.826	6.332	0.563
BL + RT + CBAM	24.177	0.866	22.981	0.843	6.532	0.583
BL + RT + LG-BPN	24.315	0.852	23.743	0.873	6.740	0.601
Full Model	**28.458**	**0.942**	**27.741**	**0.912**	**6.822**	**0.603**

## Data Availability

The data that support the findings of this study are available from the corresponding author upon reasonable request.
